# Clinical usefulness of NGS multi-gene panel testing in hereditary cancer analysis

**DOI:** 10.3389/fgene.2023.1060504

**Published:** 2023-02-01

**Authors:** Federico Anaclerio, Lucrezia Pilenzi, Anastasia Dell’Elice, Rossella Ferrante, Simona Grossi, Luca Maria Ferlito, Camilla Marinelli, Simona Gildetti, Giuseppe Calabrese, Liborio Stuppia, Ivana Antonucci

**Affiliations:** ^1^ Center for Advanced Studies and Technology (CAST), G.d’Annunzio University of Chieti-Pescara, Chieti, Italy; ^2^ Eusoma Breast Centre, “G. Bernabeo” Hospital, Ortona, Italy; ^3^ UOSD Genetica Oncoematologica, Dipartimento di Oncologico-Ematologico, Pescara, Italy

**Keywords:** NGS, hereditary cancer, BRCA, cancer predisposition gene, multi-gene panel testing, breast cancer, ovarian cancer, pancreatic cancer

## Abstract

**Introduction:** A considerable number of families with pedigrees suggestive of a Mendelian form of Breast Cancer (BC), Ovarian Cancer (OC), or Pancreatic Cancer (PC) do not show detectable *BRCA*1/2 mutations after genetic testing. The use of multi-gene hereditary cancer panels increases the possibility to identify individuals with cancer predisposing gene variants. Our study was aimed to evaluate the increase in the detection rate of pathogenic mutations in BC, OC, and PC patients when using a multi-gene panel.

**Methods:** 546 patients affected by BC (423), PC (64), or OC (59) entered the study from January 2020 to December 2021. For BC patients, inclusion criteria were i) positive cancer family background, ii) early onset, and iii) triple negative BC. PC patients were enrolled when affected by metastatic cancer, while OC patients were all submitted to genetic testing without selection. The patients were tested using a Next-Generation Sequencing (NGS) panel containing 25 genes in addition to *BRCA*1/2.

**Results:** Forty-four out of 546 patients (8%) carried germline pathogenic/likely pathogenic variants (PV/LPV) on *BRCA*1/2 genes, and 46 (8%) presented PV or LPV in other susceptibility genes.

**Discussion:** Our findings demonstrate the utility of expanded panel testing in patients with suspected hereditary cancer syndromes, since this approach increased the mutation detection rate of 15% in PC, 8% in BC and 5% in OC cases. In absence of multi-gene panel analysis, a considerable percentage of mutations would have been lost.

## 1 Introduction

In these years of personalized medicine, the study of individual’s genotype is an important part of the determination of his specific susceptibility to several diseases, including cancer. The National Comprehensive Cancer Network Breast Cancer Risk Panel (NCCN) has for years been updating with publishing the indications for genetic testing of cancer patients and their family members (Sorscher 2019). The majority of Breast Cancer (BC), Ovarian Cancer (OC) and Pancreatic Cancer (PC) cases are sporadic (75%–80%), ∼15%–20% are considered familial types and 5%–10% are hereditary (Russo et al., 2009; Antonucci et al., 2017b; Incorvaia et al., 2020). Over the past 20–30 years, molecular diagnosis of hereditary BC, OC or PC has focused primarily on two high-penetrance genes, *BRCA*1 and *BRCA*2 (Antonucci et al., 2017a). The identification of germline deleterious variants in *BRCA*1/*BRCA*2 has a significant impact on clinical management of both affected individuals and their family members (Babore et al., 2019; Lombardi et al., 2019; 2022). Nevertheless, an increasing number of families with pedigrees suggestive of a Mendelian form of BC, OC or PC have not detectable mutations in *BRCA*1/*BRCA*2. The problem of “missing heritability” can be explained with the presence of pathogenic gene variants in other susceptibility genes involved with low frequency or with reduced penetrance, usually not included in the diagnostic flowchart of patients with hereditary cancer, mainly due to the costs and the time required for the analysis in the Sanger sequencing era. Therefore, it has become mandatory to study many genes in a brief time and in an economic way. In this scenario, advances in genetic technology and implementation of NGS in clinical oncology have accelerated the discovery of new cancer-related genes revolutionizing cancer research, diagnosis and therapies (Rossi et al., 2022). The advent of NGS allows the simultaneous sequencing of multiple samples and genes (Fountzilas et al., 2018). Because of the advantage from cost-benefit reduction, this approach provides a powerful enforcement for patients with LPVs and PVs in other genes, beyond *BRCA*1/2. Several germline PVs in susceptibility genes as *CDH*1, *PALB*2, *PTEN*, STK11, *TP*53, *ATM*, *CHEK*2, *BARD*1, *BRLP*1, *RAD*51*C*, and *RAD*51*D* (Shah et al., 2016; Fanale et al., 2020) can be associated with hereditary tumors. Most of these genes are involved in cell cycle checkpoint and DNA damage repair mechanism, and function together in these physiological pathways (Nielsen et al., 2016; Piombino et al., 2020; Neiger et al., 2021); therefore, a fundamental comprehension of the disease drivers in the cascades would facilitate the accurate evaluation of the genetic risk of cancer development (Yoshimura et al., 2022). In our study we used a multi-gene panel including 27 genes in the diagnostic iter of 546 patients with BC, OC or PC (Table 1). The aims of this work were: 1) to investigate the prevalence of PVs or LPVs in susceptibility genes implicated in hereditary cancer predisposition, and 2) to assess the utility of carrying out a multi-gene panel testing in BC, OC or PC individuals who fulfill specific criteria on their familiar and personal history of tumor.

**TABLE 1:** Multi-gene panel including the 27 genes analyzed with NGS.

Multi-gene panel—next generation sequencing	
*ATM*	*BARD*1
*BRCA*1	*BRCA*2
*BRIP*1	*CDK*12
*CHEK*2	*NBN*
*PALB*2	*TP*53
*EPCAM*	*RAD*51*C*
*RAD*51*D*	*MSH*2
*APC*	*CDH*1
*CDKN*2*A*	*MKH*1
*MSH*6	*NF*1
*PMS*2	*PTEN*
*CDK*4	*MUTYH*
*POLD*1	*POLE*
*SMAD*4	

## 2 Materials and methods

### 2.1 Study population

Our study includes a cohort of individuals who referred to our Center between January 2020 and December 2021. We collected and analyzed DNA samples from 546 patients with BC (423), PC (64) or OC (59), averaging 54 years (range 25–70). For BC patients, inclusion criteria were 1) positive cancer family background, 2) early onset and 3) triple negative BC. PC patients were enrolled when affected by metastatic cancer, while OC patients were all submitted to genetic testing without selection. PC and OC patients were classified into 2 groups related to the age of disease onset: 1) early onset cancer (age at diagnosis ≤45 years) and 2) late onset cancer (age at diagnosis >45 years), while for BC patients the considered age of onset was 40 years. Among BC patients, 64 had early onset cancer and 359 had late onset cancer; among OC patients 9 had early onset cancer and 50 had late onset cancer; among PC patients 2 had early onset cancer and 62 had late onset cancer. Starting from 423 BC patients, 27 (6.4%) had triple-negative breast cancer (TNBC), including 25 patients with late onset BC and only 2 with early onset BC. Genetic counseling was performed in the presence of a geneticist and a psychologist to acquire the clinical personal and familiar history of patients. In addition, data about histological cancer type, any surgical operations and current therapies were acquired. All subjects signed an informed consent about the significance of the molecular genetic test.

### 2.2 Next-generation sequencing (NGS)

Genomic DNA of BC, OC and PC patients were collected using buccal swabs and extracted through MagPurix instrument and Forensic DNA Extraction Kit (Zinexts Life Science Corp.- CodZP01001) according to the manufacturer’s protocol. NGS was executed by the Ion Torrent S5 system (Thermo Fisher Scientific, Waltham, MA, United States) after automatic library preparation using Ion Chef (Thermo Fisher Scientific, Waltham, MA, United States). Ion Chef consists of fragmentation and adapter ligation onto the PCR products, clonal amplification. The DNA libraries were quantified with Real-Time Step One PCR System (Thermo Fisher Scientific, Waltham, MA, United States) and the prepared samples were loaded onto an Ion 530™ chip by Ion Chef (Thermo Fisher Scientific, Waltham, MA, United States). Ion S5™ Plus (Thermo Fisher Scientific, Waltham, MA, United States) instrument was used for the sequencing. Specific plugins as “SampleId” and “Coverageanalysis” were used for NGS data analysis on the Torrent Suite 5.14.0 platform. The uniformity of base coverage was over 98% in all batches, and base coverage was over ×20 at all target regions. This NGS method cannot detect variations outside the +/−10 nucleotide coding sequence.

### 2.3 Sanger sequencing

Sanger Sequencing was performed using SeqStudio Genetic Analyzer System (Thermo Fisher Scientific) and BigDye Terminator 3.1 Cycle Sequencing Kit (Thermo Fisher Scientific) to confirm all the PV/LPVs identified with NGS multi-gene panel.

### 2.4 Classification of the genetic variants

The genetic variants found in patients were classified into five classes: benign (C1), likely benign (C2), variant of uncertain significance (VUS, C3), likely pathogenic (C4), and pathogenic (C5), according to the guidelines of Evidence-based Network for the Interpretation of Germline Mutant Alleles (ENIGMA) (https://enigmaconsortium.org/). We focused on the LPVs and PVs that can be used for clinical purposes. Variants were referred in according to the nomenclature recommendations of the Human Genome Variation Society (https://www.hgvs.org). The clinical significance of the genetic variants found in this study was evaluated according to ClinVar (https://www.ncbi.nlm.nih.gov/clinvar/), Varsome (https://varsome.com), Franklin Genoox (https://franklin.genoox.com) and, for some susceptibility genes (*APC*, *MLH*1, *MSH*2, *MSH*6, *PMS*2, *EPCAM*, *MUTYH*, *CDH*1), according to LOVD-InSIGHT (https://www.insight-group.org/variants/databases/).

## 3 Results

In our study, 546 cases with BC, OC, or PC were enrolled from January 2020 to December 2021. PVs or LPVs on *BRCA*1/2 genes were detected in 44 patients (8%), specifically 32/423 (7%) with BC, 9/59 (15%) with OC and 3/64 (5%) with PC. On the other hand, 46 patients (8%), namely 33/423 (8%) with BC, 3/59 (5%) with OC and 10/64 (16%) with PC harbored germline PVs/LPVs in other cancer susceptibility genes, as follows: 17 (37%) in *MUTYH*, 11 (24%) in *CHEK*2, 4 (9%) in *ATM*, 3 (6%) in *RAD*51*C* and *TP*53, 2 (4%) *PALB*2, *BRIP*1, and *NBN*. In addition, a single PV in *POLE*, *MSH*2, and *RAD*51*D* was detected in two patients (Table 2).

**TABLE 2:** All single PVs/LPVs recurrent in patients analyzed by multi-gene panel. All variants reported in the Table 2 are in heterozygous, except only one subject that had two PVs/LPVs on MUTYH gene (*).

Gene	Refseq	Omim	HGVS Nomenclature	Protein change	Variant interpretation	Number of patients
*ATM*	NM_000051.3	607585	c.2502dup	p.(Val835fs)	PV	1 (2.1)
*ATM*	NM_000051.3	607585	c.2113del	p.(Tyr705fs)	PV	1 (2.1)
*ATM*	NM_000051.3	607585	c.756_757del	p.(Cys252_Glu253delinsTer)	LPV/PV	1 (2.1)
*ATM*	NM_000051.3	607585	c.6095G > A	p.(Arg2032Lys)	LPV/PV	1 (2.1)
*BRIP*1	NM_03204.2	605882	c.2111T > A	p.(Leu704Ter)	PV	1 (2.1)
*CHEK*1	NM_03204.2	605882	c.2392C > T	p.(Arg798Ter)	PV	1 (2.1)
*CHEK*2	NM_007194.3	604373	c.1232G > A	p.(Trp411Ter)	LPV/PV	1 (2.1)
*CHEK*2	NM_007194.3	604373	c.1100del	p.(Thr367fs)	PV	2 (4.3)
*CHEK*2	NM_007194.3	604373	c.1427C > T	p.(Thr476Met)	PV	1 (2.1)
*CHEK*2	NM_007194.3	604373	c.349A > G	p.(Arg117Gly)	LPV/PV	2 (2.1)
*CHEK*2	NM_007194.3	604373	c.409C > T	p.(Arg137Ter)	PV	1 (2.1)
*CHEK*2	NM_007194.3	604373	c.470T > C	p.(Ile157Thr)	LPV	2 (4.3)
*CHEK*2	NM_007194.3	604373	c.499G > A	p.(Gly167Arg)	LPV/PV	2 (4.3)
*MSH*2	NM_000251.2	609309	c.2647dup	p.(Ile883fs)	PV	1 (2.1)
*MUTYH*	NM_001128425.2	608456	c.1187G > A	p.(Gly396Asp))	PV	7 (15.2)
*MUTYH*	NM_001128425.2	608456	c.1437_1439del	p.(Glu480del)	PV	1 (2.1)
*MUTYH*	NM_001128425.2	608456	c.536A > G	p.(Tyr179Cys)	PV	4 (8.7)
*MUTYH*	NM_001128425.1	608456	c.1012C > T	p.(Gln338Ter)	LPV	1 (2.1)
*MUTYH*	NM_001128425.2	608456	c.734G>A (*)	p.(Arg245His)	PV	3 (10.9)
*MUTYH*	NM_001128425.2	608456	c.884C>T (*)	p.(Pro295Leu)	PV	1 (2.1)
NBN	NM_002485.4	6,026,667	c.741_742dup	p.(Glu248fs)	PV	1 (2.1)
NBN	NM_002485.4	6026667	c.2140C > T	p.(Arg714Ter)	PV	1 (2.1)
*PALB*2	NM_024675.3	610355	c.661_662delGTinsTA	p.(Val221Ter)	PV	1 (2.1)
*PALB*2	NM_024675.3	610355	c.1050_1053del	p.(Thr351fs)	PV	1 (2.1)
*POLE*	NM_006231.3	174762	c.1458delC	p.(Met487fs)	LPV	1 (2.1)
*RAD*51*C*	NM_058216.2	602774	c.1026 + 5_1026 + 7del	-	PV/LPV	1 (2.1)
*RAD*51*C*	NM_058216.2	602774	c.905-2_905-1del	-	PV	2 (4.3)
*RAD*51*D*	NM_002878.3	602954	c.898C > T	p.(Arg300Ter)	LPV/PV	1 (2.1)
*TP*53	NM_000546.5	191170	c.646G > A	p.(Val216Met)	LPV/PV	1 (2.1)
*TP*53	NM_000546.5	191170	c.637C > T	p.(Arg213Ter)	PV	1 (2.1)
*TP*53	NM_000546.5	191170	c.993G > A	p.(Gln331Gln)	PV	1 (2.1)

Seven subjects enrolled showed two pathogenic variants in the genes analyzed.

According to age of onset, we found PVs/LPVs in 20 early onset patients (≤45 for PC and OC, ≤40 for BC) and in 70 late onset patients (>45 for PC and OC, >40 for BC). Eleven early onset patients with BC (14%) had PVs or LPVs mutations in *BRCA*1 or *BRCA*2 genes, whereas 17 patients (11%) reported mutations in one of the other genes included in the multi-gene panel. On the other hand, 27 late onset patients with BC (36%) had PVs or LPVs mutations in *BRCA*or *BRCA*2 genes, whereas 30 patients (40%) reported mutations in one of the several genes included in the multi-gene panel. On the OC and PC patients groups 2 early onset subjects (18%) had a PV or LPV in *BRCA*1/2, while 2 patients (18%) had PV or LPV in other gene. In the late onset group 10 patients (9%) had a PV or LPV in *BRCA*1/2 and 11(10%) with pathogenic variant in other gene. The distribution of PVs/LPVs in *BRCA*1/2 or in other genes in the different groups of patients is reported in Table 3.

**TABLE 3:** Different groups analyzed by the age of onset criteria.

Type of tumor	BC		OC		PC	
AGE OF ONSET	≤40	>40	≤45	>45	≤45	>45
BRCA PVs/LPVs	9	23	2	7	0	3
PM PVs/LPVs	7	26	1	2	1	9
TOTAL PVs/LPVs	16	49	3	9	1	12

**FIGURE 1:**
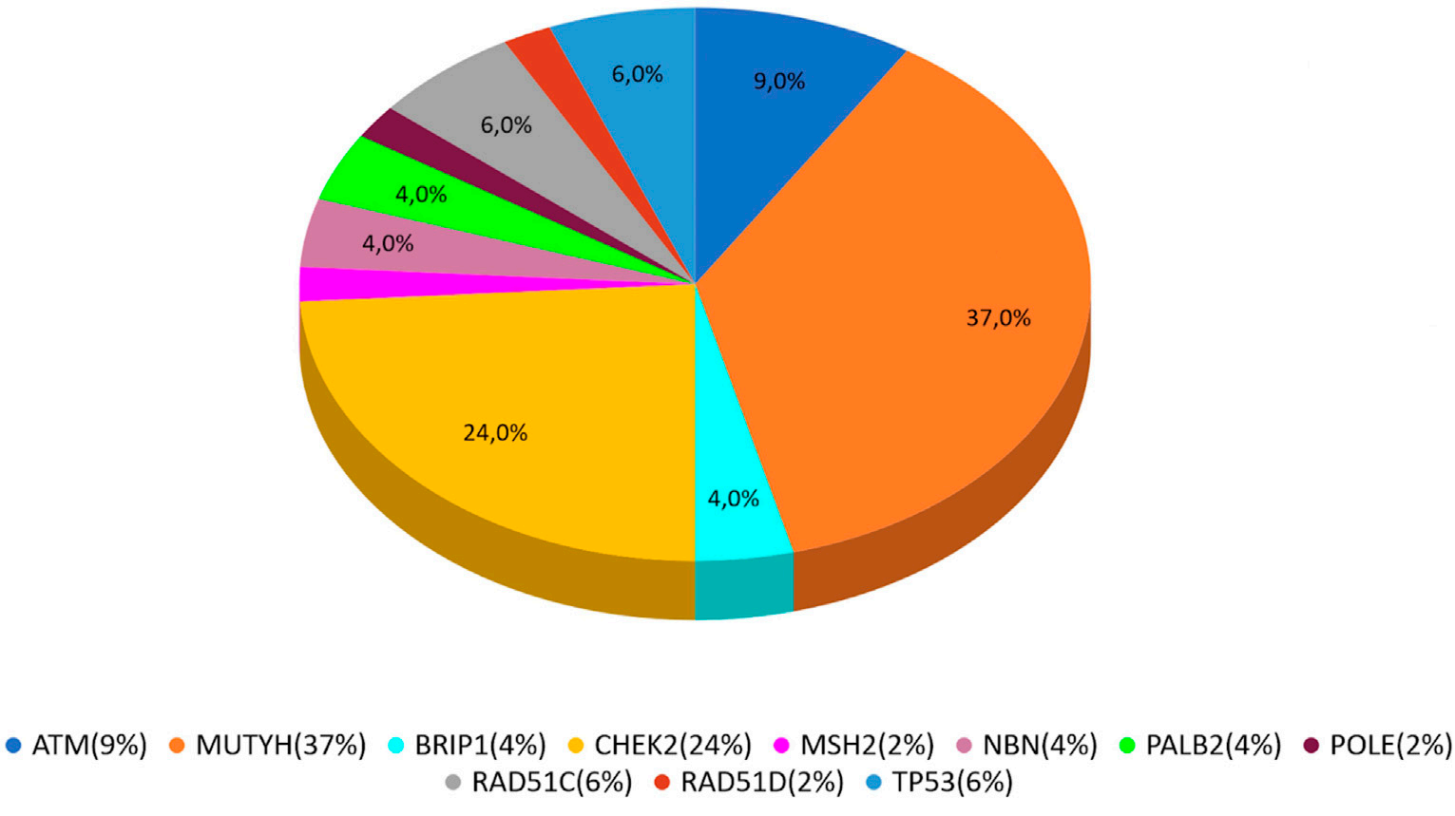
All cases analyzed with multi-gene panel.

**FIGURE 2:**
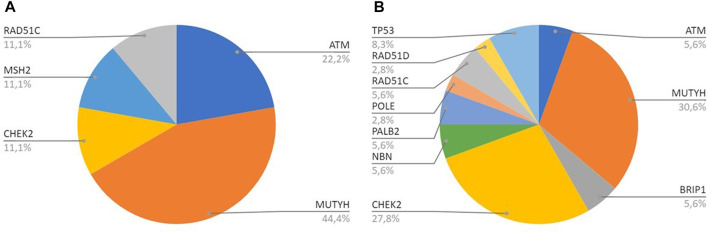
**(A)** All genes with PV/LPV in Breast Cancer cases; **(B)** All genes with PV/LPV in Pancreatic Cancer cases.

*MUTYH* resulted as the gene with the higher percentage of mutation within the group analyzed by the multigene panel (16 out of 46 detected mutations), with the second most recurrent involved genes represented by *CHEK*2 with 11 cases (Table 2; Figure 1; Figures 2A, B). All *MUTYH* variants reported in this study are in heterozygous, except only one subject that had two PVs/LPVs on *MUTYH* gene, respectively c.734G>A and c.884C>T.

As to PVs/LPVs, the most frequent PV was c.1187G>A p.(Gly396Asp) of *MUTYH* gene, located in coding exon 13 and causing the substitution of a Glycine with Aspartate in codon position 396. This alteration, found in seven patients (15.2%) with BC, PC and OC, is frequently reported as founder mutation in multiple populations. M. Nielsen et al. have shown that this missense variant change the function of *MUTYH* protein (M. Nielsen et al., 2009).

The second recurrent PV found on the *MUTYH* gene was c.734G>A p. (Arg245His), in coding exon 9, results from the substitution of a Guanine to Adenine, and consequently the replacement of the arginine with a histidine at codon 245. Literature’s data supports that this missense variant has a deleterious effect on protein structure/function (Viel et al., 2017). We found this mutation in five patients (10.9%).

Analyzing the second most gene mutated, *CHEK*2, the other most recurrent PVs were: c.1100delC p. (Thr367fs), c.470T>C p. (Ile157Thr) and c.499G>A p. (Gly167Arg). All subjects with *CHEK*2 variant, are carriers of only one PV/LPV.

Specifically, *CHEK*2 c.1100delC caused deletes of one Cytosine from exon 11 in position 1,100 causing a frameshift at codon 367, and a premature translational stop signal p. (Thr367fs). This is expected to result in an absent or disrupted protein product. (Weischer et al., 2008). This variant is linked to increased risk of BC and OC.

## 4 Discussion

The identification of PV or LPV in genes responsible for hereditary cancers plays a key role in the prognosis, prevention and therapy of these conditions. In fact, cancer patients carriers of such gene variants must undergo specific protocols for the prevention of additional cancers but they can also benefit from specific drug therapies, such as those based on PARP inhibitors (PARPi), which represent a successful example of precision medicine (Slade, 2020). On the other hand, unaffected family members of a cancer patient carrier of a PV/LPV should be tested for the presence of the same variant and, when positive, specific prevention protocols, different from the common cancer screening programs used for the general populations, should be offered. In this view, a critical issue is represented by the number of genes to analyze in each condition, mainly in order to maintain a balanced cost/benefit ratio. While in a first moment it was suggested that each different type of cancer was related to one or a few specific genes (e.g., *BRCA*1/2 for BC and OC, *APC* for familial adenomatous polyposis, etc.), our study revealed that often there is not correspondence between tumor type and the associated mutated gene, raising the question about the need for more genes to be analyzed in hereditary cancers. Interestingly, our study showed that 94% of *MUTYH* carriers had a heterozygous variant. PVs/LPVs in *MUTYH* are associated with colorectal adenomatous polyposis autosomal recessive, while recent literature data revealed the association between monoallelic *MUTYH* variants and several type of cancer (Dell’Elice et al., 2021). BC, PC and OC, together with colon and prostate cancer, are the major tumors linked to clinical familiar history, as well as the major BRCA-associated cancers (Daly et al., 2021). Nevertheless, many of these patients result negative to the genetic testing for *BRCA*1/2 genes PVs and LPVs, even in presence of an evident familiar and/or personal cancer’s background. This has been confirmed by data obtained in the present study, showing that no more that 8% of BC, OC or PC cancer show *BRCA*1/2 mutations even in the group of early onset cases.; that the use of multi-gene hereditary cancer panels increases the possibility to identify individuals with cancer predisposing gene variants (Shin et al., 2020; Hu et al., 2021). In an association analysis involving 113,000 women, the Breast Cancer Association Consortium, Dorling L, Carvalho S, et al. define the susceptibility genes that are most clinically useful for inclusion on panels for the prediction of breast cancer risk (Breast Cancer Association Consortium et al., 2021). By extending the test using a multi-gene panel, we found an additional 8% mutations in different susceptibility genes, such as *MUTYH*, *CHEK*2, *ATM*, *NBN*, *BRIP*1, and *TP*53 involved in several hereditary cancer syndromes (Desmond et al., 2015; Tsaousis et al., 2019; N; Tung et al., 2015). These results confirmed the studies already performed in 2021 by Bono et al., where a considerable percentage of PVs/LPVs have been lost without the use of multi-gene panel (Bono et al., 2021). Thus, our results evidenced that both in early and late onset cancer patients, using the classical approach of *BRCA*1/2 testing, we would have lost a large number of cases resulted *BRCA*1/2 negative, but actually carriers of a PV/LPV in other genes. In addition to the increased detection rate, the use of multigenic panel test allow the identification of specific prevention strategies based on the gene involved, in a precision medicine approach. For example, we diagnosed three patients with Li-Fraumeni syndrome (LFS) associated with PV/LPV in *TP*53 on chromosome 17p13.1. This syndrome represents a severe condition inherited in an autosomal dominant manner with very high penetrance. Prevention strategies of this condition are different from the one used for *BRCA*1/2 mutation carriers, since LFS component tumors also include soft tissue sarcomas, osteosarcoma, brain tumors, and adrenocortical carcinomas. Interestingly, in these patients no strong familiar history was found, but they all showed early onset cancer (≤35). In one case, a “*de novo*” origin of the mutation was demonstrated, allowing to suggest that the age of onset of the disease could be considered as a more reliable indicator of the presence of a genetic condition than the familiarity itself. Oncology therapy putting forth the concept of selective targeting of cancer cells thanks to precision medicine. According to our goal, one of the most interesting future perspectives is the therapy with poly-adenosine diphosphate-ribose polymerase (PARP). PARP inhibitors (PARPi) were a significant example of precision medicine (Slade, 2020). The identification of specific mutations in genes different from *BRCA*1/2 is relevant also for the therapeutical strategies. In fact, while the benefits of PARP inhibition have been well characterized for *BRCA*1/2 (Risdon et al., 2021), the efficiency of this therapy in carriers of other mutations is so far a question of debate. For the therapy of metastatic breast cancer (MBC), is in progress a phase II study that are showing the efficacy of PARPi’s Olaparib, in patients with germline/somatic (g/s) mutations in related genes (*PALB*2, *ATM* and *CHEK*2) other than *BRCA*1/2 (N. M. Tung et al., 2020). Responses were seen only with g*PALB*2 mutations, while there are not evidences for *ATM* or *CHEK*2 mutations respectively. For this reason, Olaparib could be used in patients with g*PALB*2 mutation beyond in g*BRCA*1/2 mutation carriers, significantly expanding the number of patients with MBC who would benefit from PARPi (Pommier et al., 2016; Lord and Ashworth., 2017; Cortesi et al., 2021). In conclusion, the multi-gene panel approach could be useful for targeting therapy in oncology patients that are carriers of mutations in susceptibility genes, beyond *BRCA*1/2.

## Data Availability

The datasets presented in this study can be found in online repositories. The link to the data can be found below: https://www.ncbi.nlm.nih.gov/sra/PRJNA927294. Accession to cite for these SRA data: PRJNA927294.
